# Solar flower power

**DOI:** 10.7554/eLife.33591

**Published:** 2017-12-19

**Authors:** Julia Bing, Danny Kessler

**Affiliations:** 1 Department of Molecular EcologyMax Planck Institute for Chemical EcologyJenaGermany; 2 Greenhouse DepartmentMax Planck Institute for Chemical EcologyJenaGermany

**Keywords:** pollination, temperature, angiosperms, floral signalling, infrared thermography, bumblebees, Other

## Abstract

Bumblebees use invisible temperature patterns on flowers to make foraging decisions.

**Related research article** Harrap MJM, Rands SA, Hempel de Ibarra N, Whitney HM. 2017. The diversity of floral temperature patterns, and their use by pollinators. *eLife* **6**:e31262. doi: 10.7554/eLife.31262

Most species of flowering plant cannot produce seeds without help from animals, like insects and birds, who transfer pollen between the flowers of different plants – often in return for a reward, such as a drink of sugar-rich nectar. In order to forage efficiently, pollinators seek out flowers with traits that they associate with a higher chance of getting a reward.

Some floral traits like color, shape and scent are obvious to our human senses, and as early as the 18^th^ century scientists had worked out that these signals attract insects (Sprengel, 1793). However, the majority of floral traits have been discovered just recently, using modern technology. Indeed, we now know that pollinators use many different traits to find and evaluate flowers including: CO_2_ emission (Goyret et al., 2008); ultraviolet-absorbing pigmentation (Sheehan et al., 2016); humidity surrounding the flower (von Arx et al., 2012); fluorescence (Thorp et al., 1975); nectar color (Johnson et al., 2006); and even floral temperature (Dyer et al., 2006; Whitney et al., 2008).

Bees, for example, can use heat detectors on their legs and antennae to tell the difference between two flowers that differ in temperature by just two degrees (Heran, 1952). Now, in eLife, Sean Rands and colleagues from the Universities of Bristol and Exeter – including Michael Harrap as first author – report that bumblebees can also detect temperature differences within a single flower (Harrap et al., 2017).

Thermal images of more than 100 species of flowering plant taken in sunlight revealed a wide range of temperature patterns, reminiscent of the diversity of multi-colored petals we see with our own eyes (Figure 1A). More than half of the tested species had flowers in which some parts of the petals were at least 2°C warmer than the rest. Based on these findings, the researchers hypothesized that pollinators could use these temperature patterns to decide which flowers to visit.

**Figure 1.**
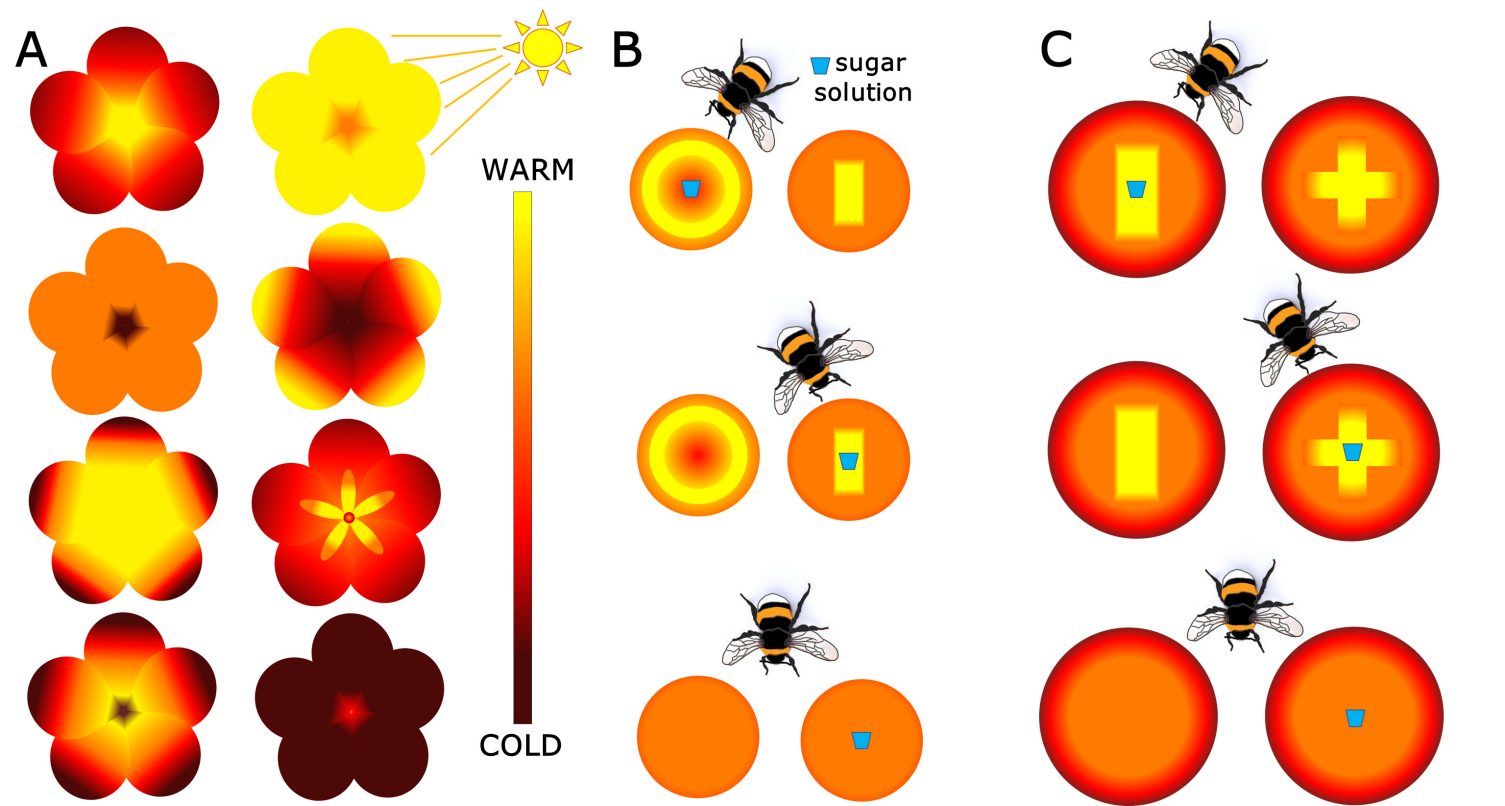
Floral temperature patterns and their role in bumblebee foraging. (**A**) Schematic examples of the diversity of floral temperature patterns in the sunlight. By using thermal imaging, Harrap et al. show that more than half of the species they tested have temperature differences within flowers that are big enough to be detected by bumblebees (color indicates temperature as shown in gradient). Experiments with (**B**) small and (**C**) large artificial flowers tested if bumblebees could associate a certain temperature pattern with a reward, in this case a sugar solution (blue cup symbol). Bumblebees were presented with two variants of temperature patterns, one containing the sugar reward and the other just water (top and middle rows). As a control, flowers without temperature patterns were used as both rewarding and non-rewarding flowers (bottom row). The position of the bumblebee in B and C shows which floral temperature pattern was visited the most after 60 visits, demonstrating that bumblebees were able to use these patterns to increase their foraging success.

Harrap et al. tested if bumblebees (*Bombus terrestris audax*) could learn to associate a reward, in this case a drop of a sugary solution, with a certain temperature pattern. They presented two types of artificial flowers containing heating elements to naïve bumblebees. The artificial flowers in the first experiment had either a warm center or a warm periphery (Figure 1B), while those in the second had warmer centers in two different shapes (Figure 1C). In all experiments, one variant contained a drop of sugar, while the other just offered water. Flowers with disconnected heating elements were used as controls.

The bumblebees did recognize different temperature patterns and, in fewer than 20 visits, had learned to forage from those flowers that would give them the reward. Importantly, when there were no temperature patterns, as is in the controls, the bumblebees could not discriminate rewarding from non-rewarding flowers. Also, once the bumblebees had learned to associate a certain temperature pattern with a sugary reward, they continued to prefer this type of flower even when the reward was removed. These results indicate that the bumblebees were using the flower temperature patterns (and not other cues) to make an informed decision when foraging.

Why did plants evolve such incredibly complex and diverse floral traits? Plants often have to compete for pollinators, either with other plant species or with other members of their own species. Any trait that enables a flower to attract more pollinators than its competitors will give it an evolutionary advantage (in other words, more seeds or higher quality offspring). Of course, plants do not invent new traits with the intention of manipulating pollinators. Instead, small mutations occur in each generation and those that change floral traits offer a chance to bring the interaction between pollinator and flower closer to perfection. Hence, it is not surprising that so many floral traits are important and act together at the same time in a single plant species.

It is exciting that sunlight is needed to turn 'on' these temperature patterns and guide pollinators to flowers. Many other floral traits are hidden from our eyes and we have just started to unveil the ways in which pollinators are able to perceive and use floral signals. Combining 'old-fashioned' natural observations with new tools, such as thermal imaging cameras or 3D printers (Campos et al., 2015), allows us to unearth the wealth of strategies that pollinators and plants use to successfully interact with each other. Floral temperature patterns triggered by sunlight to signal to bumblebees are yet another example of an impressive feat of evolution.
